# Supplementary material for: Prediction of coronary artery bypass graft outcomes using a single surgical note: An artificial intelligence-based prediction model study
Source: PLoS One. 2024 Apr 25;19(4):e0300796. doi: 10.1371/journal.pone.0300796 (PMC11045137; doi:10.1371/journal.pone.0300796)
Supplement: Attachment — Submitted filename: CABG-MN.docx [file pone.0300796.s001.docx]

I would like to thank the authors for studying the outcomes following CABG using AI approaches. It is an interesting paper. However, there are some comments as follows:

Title: It is recommended to change the title to “Prediction of Coronary Artery Bypass Graft Outcomes Using a Single Surgical Note: An Artificial intelligence-based prediction model study”

**Introduction:**

Line 82-83: It is recommended to explain the superiority of AI to conventional analysis (e.g., logistic regression). To the best of my knowledge, AI models did not perform better. Please explain the statistical rational for this approach.

Line 84: In prediction model studies, it is better use predictors instead of risk factors. Please consider it in the whole manuscript, as well.

**Methods:**

This section has been written well. But, according to the recent recommendation, it is recommended to write this section based on TRIPOD writing standard to improve the study replicability.

Please identify that isolated on-pump or off-pump CABG?

Line 195, SHAP value? Please provide its expanded form.

Line 119-120 Performance was measure by AUC. AUC provides information on discriminative ability of the models. It is recommended to provide the Calibration of the models, as an Achill heel in prediction model studies.

Please provide IRB or REC approval.

**Results:**

line 212: median of AUC. It is better to use average instead.

**Table 1:** It is recommended to write Table 1 in a way to be understand easily by the readers. Please provide the expanded form of abbreviations bottom of the table.

**Discussion:**

Please discuss the results with previous studies in terms of AUC and predictors.

You can use the following large scale studies:

- Nomali, M., Heidari, M.E., Ayati, A. *et al.* Risk factors of in-hospital mortality for isolated on-pump coronary artery bypass graft surgery in the northeast of Iran from 2007 to 2016. *Ir J Med Sci* (2023). <https://doi.org/10.1007/s11845-023-03298-6>
- Sattartabar B et al (2021) Sex and age difference in risk factor distribution, trend, and long-term outcome of patients undergoing isolated coronary artery bypass graft surgery. BMC Cardiovasc Disord 21(1):1–10

Also, please provide study limitations and implication.
