# Supplementary material for: Prediction of coronary artery bypass graft outcomes using a single surgical note: An artificial intelligence-based prediction model study
Source: PLoS One. 2024 Apr 25;19(4):e0300796. doi: 10.1371/journal.pone.0300796 (PMC11045137; doi:10.1371/journal.pone.0300796)
Supplement: Attachment — Submitted filename: response.docx [file pone.0300796.s002.docx]

Author Response to Review Comments

**Reviewer 1**

*I would like to thank the authors for studying the outcomes following CABG using AI approaches. It is an interesting paper. However, there are some comments as follows:*

*Title: It is recommended to change the title to “Prediction of Coronary Artery Bypass Graft Outcomes Using a Single Surgical Note: An Artificial intelligence-based prediction model study”*

The title has been changed accordingly.

*Introduction:*

*Line 82-83: It is recommended to explain the superiority of AI to conventional analysis (e.g., logistic regression). To the best of my knowledge, AI models did not perform better. Please explain the statistical rational for this approach.*

Thank you for the suggestion. We now include a sentence in both the introduction and discussion mentioning that the achieved AUCs are higher than the STS risk calculator’s conventional analysis, and that the AI techniques are suitable for automation in comparison to the current standard’s manual data entry. We reference other analyses that achieve similar or even slightly superior performance from structured data than our analysis. We point out that these analyses utilize structured data, and that an AI model that combines unstructured data, such as notes, with the structured data can likely surpass either modality alone. We point out that the model performances indicate that both the notes and structured EHR data contain predictive signal.

*Line 84: In prediction model studies, it is better use predictors instead of risk factors. Please consider it in the whole manuscript, as well.*

Thank you for noting this. We replaced “risk factors” with “predictors.”

*Methods:*

*This section has been written well. But, according to the recent recommendation, it is recommended to write this section based on TRIPOD writing standard to improve the study replicability.*

Methods have been updated to the best of my ability according to the TRIPOD checklist.

*Please identify that isolated on-pump or off-pump CABG?*

About 5.4% of patients at this institution are off-pump. This information has been included in the manuscript.

*Line 195, SHAP value? Please provide its expanded form.*

We updated the document to correct for missing this: “we calculate SHAP (Shapley Additive exPlanation) values at an Ngram resolution (SHAP-N) …”

*Line 119-120 Performance was measure by AUC. AUC provides information on discriminative ability of the models. It is recommended to provide the Calibration of the models, as an Achill heel in prediction model studies.*

We added a calibration analysis via a linear regression with validation set predictions as the dependent variable and observed event rate as the y-axis. The fit linear transformation is then applied to the holdout set predictions.

*Please provide IRB or REC approval.*

We added that “the study was deemed exempt by the Medical University of South Carolina institutional review Board (IRB Pro00122587)” to the end of the Data sub-section of “Materials and Methods.”

*Results: line 212: median of AUC. It is better to use average instead.*

Thank you, it now states the mean.

*Table 1: It is recommended to write Table 1 in a way to be understand easily by the readers. Please provide the expanded form of abbreviations bottom of the table.*

The abbreviations have been expanded.

*Discussion:*

*Please discuss the results with previous studies in terms of AUC and predictors.*

*You can use the following large scale studies:*

*Nomali, M., Heidari, M.E., Ayati, A. et al. Risk factors of in-hospital mortality for isolated on-pump coronary artery bypass graft surgery in the northeast of Iran from 2007 to 2016. Ir J Med Sci (2023).* [*https://doi.org/10.1007/s11845-023-03298-6*](https://doi.org/10.1007/s11845-023-03298-6)

*-Sattartabar B et al (2021) Sex and age difference in risk factor distribution, trend, and long-term outcome of patients undergoing isolated coronary artery bypass graft surgery. BMC Cardiovasc Disord 21(1):1–10*

We now include references to these two papers in the discussion. Specifically, that (1) these previous studies identify correlated features with risk of mortality; (2) that the AI model achieves higher AUC; (3) however, AI that incorporates the structured features from the previous analyses with unstructured data can likely achieve superior performance than AI on the note alone.

*Also, please provide study limitations and implication.*

This is now part of the discussion section.

**Reviewer 2**

*Sherard et al. have conducted a study on designing AI models for the prediction of CABG outcomes in a single center. The findings are interesting; however, there are concerns that should be addressed.*

Thank you for your time reviewing our manuscript.

*Features used for training the models should be more explained since they are not clear to the readers. What do the authors mean by surgical notes in the methods and also feature importance in the results section?*

We agree. We added information about the notes in the data sub-section of methods, that they are consult notes and present a somewhat common format found in the notes. The features are presented in more details in the Methods section, specifically how the features correspond to ngrams that contribute to model prediction.

*The abbreviations should be mentioned in complete form in their first use (e.g., IQR).*

Thank you, this has been updated in the manuscript.

*The headings “Permutation Tests”, “Embedding Correlations”, and “Feature Importance” in the results section should be further elaborated.*

The headings now have more descriptive names. Thank you for pointing this out.

*In the discussion section, a comparison should be made with other AI studies performed on patients undergoing CABG (such as 10.3389/fcvm.2022.977747, 10.3389/fcvm.2022.881881, and 10.1016/j.athoracsur.2021.08.040)*

Thank you, we now mention that these studies with structured EHR data achieve similar or even superior performance to our own analysis in the discussion section. However, due to the high performance of the AI model on unstructured note text, we hypothesize that a model which combines the structured data in previous studies with AI processing of unstructured data such as this analysis can achieve higher performance than either modality alone.

*The study lacks a limitation section which should be mentioned before the conclusion.*

A limitation section has been added to the discussion.

**Reviewer #3**

*Summary: The authors have presented two Convolutional Neural Network (CNN)-based models designed to predict Coronary Artery Bypass Grafting (CABG) outcomes utilizing a single pre-operative surgical note. The first model, TextCNN, is a previously known CNN architecture specifically tailored for processing textual input, while the second model (AttnToNumCNN) represents an enhanced CNN architecture that incorporates a novel embedding pipeline for encoding numerical values. Furthermore, the enhanced model incorporates a multi-headed self-attention module. Notably, the authors have demonstrated good predictive performance with Area Under the Receiver Operating Characteristic Curve (AUC) values of 0.79 and 0.82 for TextCNN and the enhanced model, respectively.*

*Strengths:*

** New numerical data embedding pipeline: The introduction of a new embedding layer for encoding numerical data within the medical context is a notable contribution.*

** Performance on the primary task: The study has successfully achieved strong predictive performance on the primary task, as evidenced by the AUC values, underscoring the effectiveness of the proposed models in predicting CABG outcomes.*

** SHAP Value Utilization: The incorporation of SHAP (Shapley additive explanations) values to explain the model's predictions is a commendable approach, fostering interpretability and transparency in the model's decision-making process.*

*Weaknesses:*

** Lack of Clarity Regarding New Modules: The manuscript could benefit from a more explicit presentation of the specific contributions and impacts of the novel modules introduced in the enhanced model.*

This suggestion greatly expanded and improved our research. We added an ablation analysis that compares the basic model which just tokenizes all numbers as words (BASIC), the advantage of only tokenizing numbers within context of keywords (remove out-of-context numbers, ROOC), the advantage of the numerical scaling technique (ScaleNum), and finally the advantage of the attention-based embedding technique applied to the number embeddings (AttnToNum).

** Limited Model Exploration: The authors have primarily explored CNN-based architectures in this study. However, considering the sequential nature of medical notes, it would be advantageous to experiment with alternative models, such as Long Short-Term Memory (LSTM) or Transformer-based architectures, which are designed for effective long-sequence processing.*

Thank you, we have included results for bi-directional LSTM, and listed as a limitation that we did not run these analyses on LLMs and other transformer models due to scope of the work. This suggestion led to more evidence of the utility of our numerical embedding techniques as the same pattern of improvement was found with the LSTM architecture.

Issues

Major:

1. *The authors have incorporated two novel modules, namely the embedding layer and the self-attention module, into their AttnToNumCNN architecture. They subsequently compare its performance to the baseline TextCNN model. However, a key issue arises in disentangling the distinct contributions of each of these modules to the observed performance enhancement. This lack of clarity raises the possibility that the improved performance may be primarily attributed to the introduction of the self-attention module alone, even if used along with the original embedding layers of the TextCNN model. Furthermore, the introduction of both the embedding layer and self-attention module introduces additional trainable parameters into the enhanced architecture, thereby complicating the fair comparison with the TextCNN model. To address this concern, it is advisable for the authors to report the total number of parameters for both models (it would be great if the number of parameters of the new modules are reported separately as well) and perform an ablation study by introducing only the new embedding layer, subsequently reporting its net impact on performance and then doing the same after adding the self-attention layer. This approach would help disentangle the individual contributions of these modules and enhance the interpretability of the performance improvements observed.*

This is a great idea. We therefore now include the performance advantage of each of the contributions between basic tokenization, to removing numbers not near context words, to scaling the numerical values within context, to finally applying context to the numerical embeddings with AttnToNum. We present the number of parameters associated with each embedding technique.

*Minor:*

1. *Utilize Alternative Text Models: While the authors justified their omission of larger models like BERT due to computational costs and literature suggesting comparable performance with smaller models, it is advisable to explore traditional text-processing model families such as RNN and LSTM. These architectures are better suited for processing long-sequences compared to CNNs, which mainly focus on local patterns. Additionally, considering the dominance of Transformer-based architectures in NLP tasks, incorporating a scaled-down Transformer variant with a comparable parameter count to TextCNN could provide valuable insights into their relative effectiveness.*

In response to these requests, we now include LSTM. We agree that an analysis which compares transformer performance is highly valuable. Due to our current word count and paper breath, we aim to run a thorough analysis with LLMs in follow-up research.

2. *Visualize Self-Attention Weights: It is recommended to visualize the self-attention weights for a few input examples to gain visual insights into the behavior of the attention layer. This visualization would enhance the comprehensibility of the attention mechanism's functioning, thereby contributing to a more thorough investigation of its role in model decisions.*

Thank you and we agree. Example phrases with attention weights are now presented in Table 4.
