# Supplementary material for: Prediction of coronary artery bypass graft outcomes using a single surgical note: An artificial intelligence-based prediction model study
Source: PLoS One. 2024 Apr 25;19(4):e0300796. doi: 10.1371/journal.pone.0300796 (PMC11045137; doi:10.1371/journal.pone.0300796)
Supplement: Attachment — Submitted filename: response.docx [file pone.0300796.s003.docx]

Author Response to Review Comments

**Reviewer 1**

*I would like to thank the authors for dedicating time to revise the paper accordingly. However, there are comments as follows:*

*Abstract, methods: Please provide the expanded form of MM at first.*

The abstract methods section has been changed accordingly.

*Abstract, results: I could not understand why you provided IQR for ROC instead of 95% CI. Please provide AUC and its uncertainty (95%CI).*

This is a good point. We now report the mean AUC and the 95% CI.

*Introduction:*

*Please provide the expanded form of AI, firstly. Please consider it in the whole document.*

The first mentions of AI in the Introduction, Results, and Abstract sections are now expanded first.

*How about Research ethics approval? And, calibration as a performance measure for prediction model studies?*

We clarified that “the study was deemed exempt from review by the institutional review Board (IRB Pro00122587), *the committee who oversees research ethics at the Medical University of South Carolina*” in the end of the Data sub-section of “Materials and Methods”.

To address calibration as a performance measure, we have added the sentence: “Our results indicate that low parameter-count neural networks can achieve superior predictive performance to the current risk calculator standard, given utilization of the novel numerical embeddings and calibration of model output to a desired class-separation threshold.”

*Results:*

*You can not report mean and IQR. You can report Mean and SD or Median and IQR. In addition, you must report AUC and 95 % Confidence interval, instead.*

We now report mean, SD, and 95% confidence intervals.

*This section has been written in a complex way that may be it is interested for statistician or epidemiologist NOT clinicians. It recommended to use graph or table to facilitate its understanding.*

Thank you for this comment. We have added three additional tables to improve feasibility of result visualization to this section.

Thank you for your time and these helpful revisions. They greatly improved the quality of the paper in our opinion. We hope these updates addressed the reviewer concerns.

John Del Gaizo and Curry Sherard
